# Alterations in peripheral blood memory B cells in patients with active rheumatoid arthritis are dependent on the action of tumour necrosis factor

**DOI:** 10.1186/ar2718

**Published:** 2009-06-05

**Authors:** M Margarida Souto-Carneiro, Vijayabhanu Mahadevan, Kazuki Takada, Ruth Fritsch-Stork, Toshihiro Nanki, Margaret Brown, Thomas A Fleisher, Mildred Wilson, Raphaela Goldbach-Mansky, Peter E Lipsky

**Affiliations:** 1 Centro de Neurociências e Biologia Celular, Department of Zoology, University of Coimbra, 3004-517 Coimbra, Portugal; 2 National Institute of Arthritis and Musculoskeletal and Skin Diseases, NIH, 9000 Rockville Pike, Bethesda, MD 20892, USA; 3 Department of Medicine and Rheumatology, Graduate School, Tokyo Medical and Dental University, 1-5-45, Yushima, Bunkyo-ku, Tokyo 113-8519, Japan; 4 Department of Rheumatology, UMC Utrecht, Heidelberglaan 100, 3584 CX Utrecht, The Netherlands; 5 Department of Laboratory Medicine, Warren Magnuson Center, NIH, 9000 Rockville Pike, Bethesda, MD 20892, USA

## Abstract

**Introduction:**

Disturbances in peripheral blood memory B cell subpopulations have been observed in various autoimmune diseases, but have not been fully delineated in rheumatoid arthritis (RA). Additionally, the possible role of tumour necrosis factor (TNF) in regulating changes in specific peripheral blood memory B cell subsets in RA is still unclear.

**Methods:**

The frequency and distribution of B cell subsets in the peripheral blood and synovial membrane of active RA patients with long-standing disease have been analysed. Additionally, the possible role of TNF in causing disturbances in memory B cell subsets in RA patients was assessed in a clinical trial with the specific TNF-neutralising antibody, infliximab.

**Results:**

RA patients, independent of disease duration, have a significantly lower frequency of peripheral blood pre-switch IgD^+^CD27^+ ^memory B cells than healthy individuals, whereas post-switch IgD^-^CD27^+ ^accumulate with increased disease duration. Notably, both pre-switch IgD^+^CD27^+ ^and post-switch IgD^-^CD27^+ ^memory B cells accumulate in the synovial membrane of RA patients. Finally, anti-TNF therapy increased the frequency of pre-switch IgD^+^CD27 memory B cells in the peripheral blood.

**Conclusions:**

The data suggest that decreases in peripheral blood IgD^+^CD27^+ ^pre-switch memory B cells in RA reflect their accumulation in the synovial tissue. Moreover, the significant increase in the peripheral blood pre-switch memory B cells in patients who underwent specific TNF-blockade with infliximab indicates that trafficking of memory B cells into inflamed tissue in RA patients is regulated by TNF and can be corrected by neutralising TNF.

## Introduction

Rheumatoid arthritis (RA) is a chronic systemic autoimmune disease, characterised by inflammatory polyarthritis and joint damage resulting in progressive disability [1]. The inflammatory infiltrate in RA includes T cells, B cells and dendritic cells [2-4], and in approximately 20% of patients lymphoid neogenesis develops with the formation of ectopic germinal centres [5-8].

The importance of B cells in RA has been emphasised by the success of therapeutic approaches using anti-CD20 monoclonal antibodies (mAbs) [9]. It is currently unknown whether this approach to treatment is successful because of the production of early plasma cells due to the loss of rheumatoid factor or because of other functions of B cells.

Functionally distinct B cell subsets can be defined by the surface expression of immunoglobulin (Ig) D and CD27. These include naïve IgD^+^CD27^-^; pre-switch memory IgD^+^CD27^+^; and post-switch memory IgD^-^CD27^+ ^[10-12]. Importantly, CD27 expression by B cells has been considered a hallmark for cells that have undergone somatic hypermutation [13], although recently a CD27^- ^population of memory B cells with mutated Ig genes has been described [14-16], which is elevated in patients with systemic lupus erythematosus (SLE) [15]. Abnormalities in the frequencies of peripheral blood memory B cells have been reported in SLE [17], and Sjögren's syndrome (SS) [18]. However, in RA the data on possible disturbances of peripheral blood B cell distributions have not been delineated as well. Part of this could relate to differences in disease duration and therapy of the cohorts studied [19-21].

Treatment with TNF blockers ameliorates the signs and symptoms of RA and disease progression [22-25]. Recently, a study of peripheral blood and tonsilar biopsies from RA patients undergoing treatment with the combined TNF and lymphotoxin α (LTα) antagonist, etanercept, suggested that part of the success of this therapy in RA could be linked to a disruption of follicular dendritic cell (FDC) networks in secondary lymphoid organs, thus impairing germinal centre formation, and decreasing the number of CD27^+^memory B cells in the blood [19]. However, this effect was noted in the tonsil, making it uncertain whether etanercept would have a similar impact on germinal centres in the spleen and lymph nodes. Etanercept neutralises both TNF and LTα, so it is difficult to determine the possible contribution of each cytokine to the effects noted. TNF and LTα have many non-overlapping functions and, therefore, distinct effects of blocking each of these two cytokines on memory B cell homeostasis are possible. For example, TNF is involved in the regulation of the expression of adhesion molecules, such as vascular cell adhesion molecule (VCAM-1), intercellular adhesion molecule (ICAM-1), P-selectin, E-selectin, and L-selectin (reviewed in [26]) and also vascular endothelial growth factor (VEGF)-C [27], suggesting that it may play a crucial role in the neovascularisation of rheumatoid synovium and also recruitment of lymphocytes into the inflamed synovium.

In order to study the changes in peripheral memory B cell subpopulations in RA patients, and to understand the possible role of TNF in regulating changes in specific memory B cells, we analysed the frequency and distribution of B cell subsets in the peripheral blood and synovial membrane of active RA patients with long-standing disease. Subsequently, we assessed whether treatment with the specific TNF-blocker, infliximab, normalised the distribution of these peripheral B cell subsets. Our results show, for the first time, that RA patients, independent of disease duration, have a much lower frequency of peripheral blood pre-switch IgD^+^CD27^+ ^memory B cells than healthy individuals, whereas post-switch IgD^-^CD27^+ ^memory B cells accumulate with increased disease duration. Additionally, we present evidence that pre-switch IgD^+^CD27^+ ^memory B cells accumulate in the synovial membrane of RA patients, and that this accumulation might be related to the influence of TNF, because anti-TNF therapy increased the frequency of pre-switch IgD^+^CD27 memory B cells in the peripheral blood. These results document disease-related and TNF-dependent abnormalities in memory B cell subsets in RA and suggest that part of the success of TNF neutralising therapy could relate to normalisation of memory B cell abnormalities.

## Materials and methods

### Patients and controls

Peripheral blood samples from 40 healthy donors (26 females, 14 males; mean age 44 years) were obtained from the National Institutes of Health blood bank, and from 33 patients (28 females, five males; mean age 57 years) with long-standing RA (median disease length, 13 years) enrolled in a natural history protocol (00-AR-0222) at the Warren G. Magnuson Clinical Center (National Institutes of Health, Bethesda, Maryland, USA).

In addition, blood samples were obtained from 23 patients (20 females, 3 males; mean age 48.5 years) with active RA (defined as having greater than four tender and swollen joints, erythrocyte sedimentation rate (ESR) greater than 20 mm/hour or C-reactive protein (CRP) greater than 0.8 mg/dl) who failed treatment with methotrexate (MTX; 12.5 to 15 mg/week) and were entering a clinical trial of infliximab therapy (00-AR-0220). For this trial, patients on prednisone had to be on 7.5 mg or less per day to be eligible to participate. Patients were randomised to receive either monthly infliximab infusions (3 mg/kg infliximab with MTX 15 mg/week), or monthly control infusions and weekly MTX alone (<25 mg/week). All patients fulfilled the revised American College of Rheumatology criteria for RA [28]. MB, carrying out the flow cytometric analysis, was blinded to the measurements of clinical response and disease activity scores.

The group of patients enrolled in the natural history protocol, with a median disease length of 13 years, were considered as the long-standing disease group. The group of patients enrolled in the clinical trial for infliximab, with a median disease duration of 4.4 years, were considered as the group with shorter disease duration.

Synovial specimens and peripheral blood samples were collected at the Department of Rheumatology, Tokyo Medical and Dental University from 10 RA subjects with long-standing disease (median disease length of 13.5 years).

The characteristics of all patients studied are shown in Table 1.

**Table 1:** Clinical and demographic characteristics of the RA patients

				Treatment trial	
				
	Healthy controls	Long-standing RA	RA patients for synovium collection	MTX only	Infliximab and MTX
Number of subjects	40	33	10	8	15
Age (years)	44 ± 9	57 ± 12	62 ± 10	52 ± 16	45 ± 11
Female/male ratio	26:14	28:5	9:1	7:1	13:2
Disease duration (years)	--	13 ± 12	13.5 ± 11.0	5.7 ± 1.5	3.0 ± 0.5
% RF positive patients	--	79%	90%	50%	73%
ESR (mm/hour)	--	36 ± 27	--	36.9 ± 14.3	60.2 ± 30.3
CRP (mg/dl)	--	<0.4 ± 9.75	2.8 ± 2.3	0.8 ± 1.1	1.8 ± 1.9
% Patients on MTX (dose)	--	85% (15 mg/wk)	60% (4 mg/wk)	100% (14 mg/wk)	100% (14 mg/wk)
% Patients on GC (dose)	--	49% (5 mg/day)	80% (5 mg/day)	50% (6 mg/day)	64% (6 mg/day)
% Patients on other DMARD	--	82%	50%	--	--

Data are means ± standard deviation.

CRP = C-reactive protein; DMARD = disease-modifying anti-rheumatic drug; ESR = erythrocyte sedimentation rate; GC = glucocorticoids; MTX = methotrexate; RA = rheumatoid arthritis; RF = rheumatoid factor.

The local institutional review board or the ethics committees (National Institutes of Health and Tokyo Medical and Dental University) approved the studies and all patients signed an informed consent before participating in this study. Patient's management was performed in accordance with the local standard practice and the study was conducted in accordance with the regulations governing clinical trials, such as the Declaration of Helsinki as amended in Edinburgh (2000).

### Lymphocyte phenotyping

#### Peripheral Blood

Peripheral blood samples from the controls and natural history patients were obtained during a single scheduled outpatient visit. Peripheral blood mononuclear cells (PBMCs) were isolated by Ficoll gradient centrifugation and re-suspended in 1.5 ml PBS and 1% BSA (1 × 10^6 ^cells/100 μL). Isolated PBMCs were stained by standard methods with fluorescein isothiocyanate (FITC), phycoerythrin (PE), peridinin-chlorophyll-protein Cy5.5 (PerCpCy5.5) or allophycocyanin (APC) conjugated mAb specific for the following human cell surface markers: anti-CD19 PerCpCy5.5, anti-CD27 PE, anti-IgD FITC and anti-IgM FITC (all mAb were obtained from BD Pharmingen, Franklin Lakes, NJ, USA). Data were acquired on a FACSCalibur (BD Biosciences, Franklin Lakes, NJ, USA).

Peripheral blood samples from the RA patients treated with MTX and infliximab or MTX alone were obtained before and after treatment. Anticoagulated samples were stained for three-colour flow cytometry using a whole blood staining method at the National Institutes of Health Clinical Center laboratory. B cells were identified by staining with anti-CD20 APC and anti-CD27 PE (BD Biosciences, San Jose, CA, USA) and anti-IgD FITC (Caltag, Burlingame, CA, USA). T cells were identified by anti-CD3 APC or PE, anti-CD4 PE, anti-CD8 FITC or APC, anti-CD45RA FITC and anti-CD45R0 APC (BD Biosciences, San Jose, CA, USA).

To calculate absolute numbers of each lymphocyte subset, the percentage of cells staining positively was multiplied by the absolute peripheral blood lymphocyte count, which was determined by cell counting with a Celldyne 3500 (Abbott, Santa Clara, CA, USA) blood cell counting machine. With all experiments, peripheral blood from healthy adult patients was stained and analysed as controls.

To determine the chemokine receptor expression by B cells and their subsets, the following APC-conjugated anti-human mAbs were used: anti-CXCR1, anti CXCR2 and anti-CCR2 (R&D Systems, Minneapolis, MN, USA); and anti-CXCR4 (BD Biosciences, San Jose, CA, USA).

Irrelevant, directly conjugated, murine IgG1 (BD Biosciences, San Jose, CA, USA) was used to ascertain background staining. Samples were run on a FACScan or a FACSCalibur (BD Biosciences, San Jose, CA, USA). Data were analysed using the WinList software, version 5.0, and FloJo software (TreeStar, Stanford University, CA, USA). B cells (CD20^+ ^or CD19^+^) were gated and the percentages of CD27^+ ^(total memory), IgD^+^CD27^- ^(naïve), IgD^+^CD27^+ ^(pre-switch memory) and IgD^-^CD27^+ ^(post-switch memory) populations in the gated B cells were calculated. Although anti-CD20 mAb do not identify all plasmablasts, most of which are CD19^+^CD27^++^IgD^-^, the results from both staining protocols were pooled together, because no significant differences in total or post-switch memory B cells were observed when analysing the results separately.

T cells (CD3^+^) were gated, and the precentages of CD4^+ ^(total helper), CD8^+ ^(total cytotoxic), CD4^+^CD45RA^+ ^(total naïve helper) and CD4^+^CD45R0^+ ^(total memory helper) populations within the T cell population were calculated.

#### Synovial specimens

Synovial tissues were obtained during joint replacement surgery from 10 RA patients. Specimens were minced and incubated with 0.3 mg/ml of collagenase (Sigma, St. Louis, MO, USA) for one hour at 37°C in Dulbecco's Modified Eagle's Medium (DMEM; Sigma, St. Louis, MO, USA). Partially digested pieces of the tissue were pressed through a metal screen to obtain single cell suspensions. Cells were stained with anti-CD19 PECy5 (Beckman Coulter, Fullerton, CA, USA), anti-CD27 FITC, anti-IgM PE and anti-IgD PE (all from Becton Dickinson, Fullerton, CA, USA), anti-CXCR1 PE, anti-CXCR2 PE, anti-CXCR4 PE and anti-CCR2 PE (all from R&D Systems Inc., Minneapolis, MN. USA). Synovial tissue cells were adjusted to 1 × 10^5 ^cells, and incubated with the above mAbs for 30 minutes, rinsed with PBS-3% FCS, and analysed with a FACSCalibur (Becton Dickinson, Fullerton, CA, USA).

### Amplification of the IgV heavy chain by single-cell PCR

CD19^+^IgD^+^CD27^- ^naïve B cells and CD19^+^IgD^+^CD27^+ ^memory B cells from four patients with RA were sorted using a Beckton Dickinson FACS DIVA (Fullerton, CA, USA) or a Dako Cytomation MoFlo (Dako Cytomation, Ft Collins, CO, USA) and 1 to 1.5 cells/5 μL PBS, and then plated into 96-well PCR plates containing 10 μL lysis buffer (2 × PCR buffer + 0.4 mg/ml proteinase K (Sigma, St. Louis, MO, USA)), subjected to primer extension pre-amplification and then VH3 and VH4 genes were amplified by nested PCR, as previously described [29]. PCR products were purified using the Performa^® ^96-Well Standard Plate kit (Edge BioSystems, Gaithersburg, MD, USA) and sequenced on a model 3100 capillary sequencer (Applied Biosystems, Foster City, CA, USA) using the Big Dye^® ^Terminator v1.1 Cycle Sequencing Kit (Applied Biosystems, Foster City, CA, USA). Ig variable heavy chain rearrangements were analysed for somatic mutations using the web-based algorithm JOINSOLVER^® ^(NIAMS/CIT, Maryland, USA) [30].

### Soluble CD27 ELISA

The level of soluble CD27 was determined in serum samples from RA patients in the natural history protocol and healthy controls using the PeliKline Compact human soluble CD27 ELISA kit (CLB, Central Laboratory of the Netherlands Red Cross, Amsterdam, The Netherlands) according to the manufacturer's instructions.

### Statistical analyses

Data were checked for a normal distribution in order to decide whether to use parametric or non-parametric tests. Median group values (with standard error of the mean) for percentage and absolute numbers of the different B cell populations were compared in patients and healthy controls using the nonparametric unpaired Mann-Whitney test.

Mean values (with standard deviation) of the CD27^+ ^memory B cell population were compared between the synovium and peripheral blood of 10 RA patients undergoing synovectomy using a paired Student's t-test.

Median group values (with standard error of the mean) of the different B cell populations compared pre- and post-treatment in the 23 RA patients who were treated with infliximab plus MTX or MTX monotherapy using the nonparametric paired Wilcoxon Signed Rank test. A *P *< 0.05 was considered statistically significant.

## Results

### Characteristics of the RA patients

The demographic and clinical characteristics of the RA patient groups evaluated in this study are shown in Table 1. Most of the 33 patients with long-standing RA were women with chronic (median disease duration of 13 years), rheumatoid factor-positive erosive disease. All patients were receiving MTX alone or in combination with other disease-modifying anti-rheumatic drugs (DMARDs). Most of the subjects from whom synovial specimens were obtained were also older women with chronic rheumatoid factor-positive RA.

The 23 RA patients enrolled in a clinical trial comparing MTX plus infliximab with MTX alone had disease of shorter duration (median 4.4, infliximab + MTX: 3.0 and MTX: 5.7 years).

### RA patients have a reduced peripheral blood pre-switch IgD^+^CD27^+ ^memory B cell population

**Figure 1:**
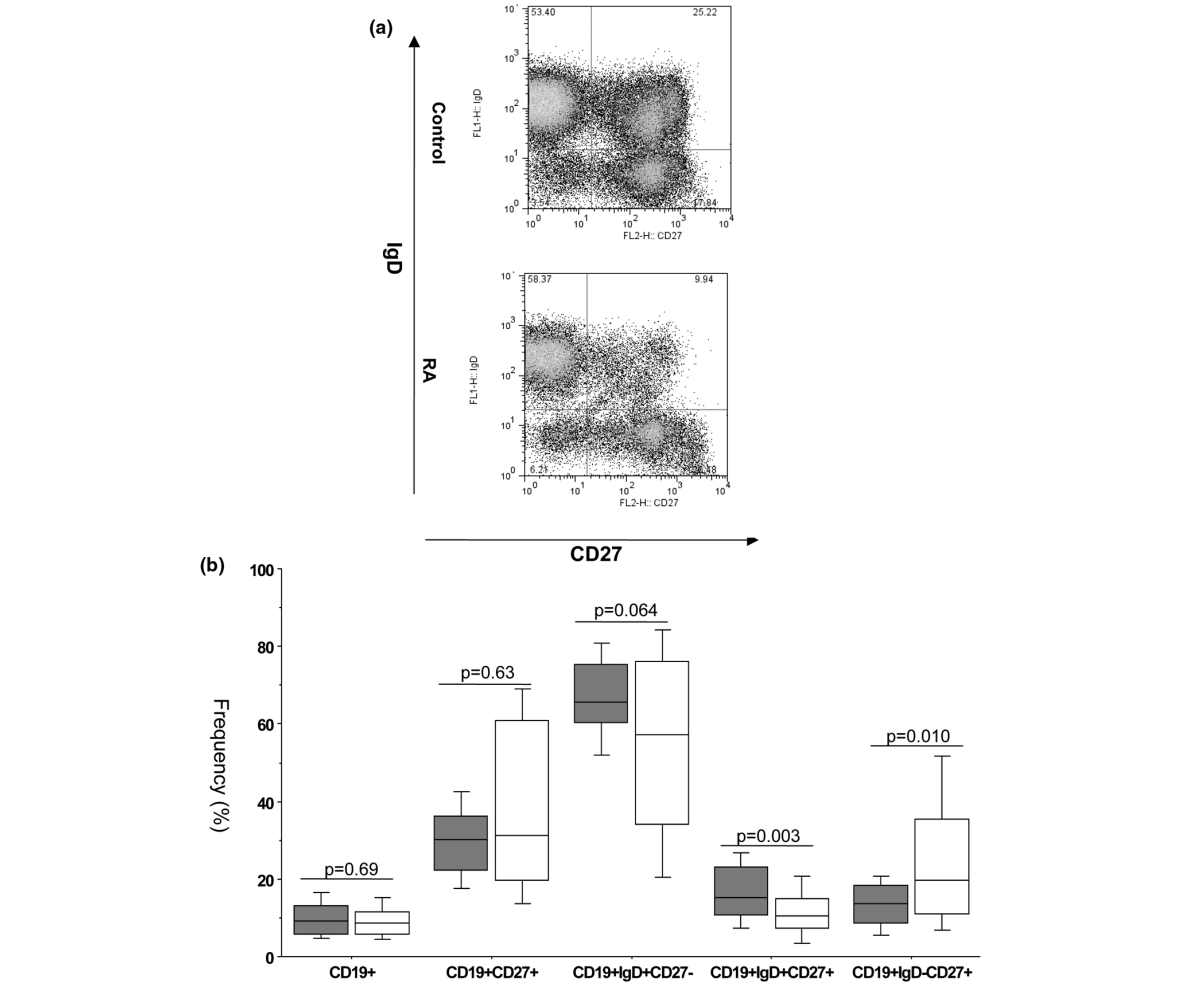
RA patients, irrespective of disease duration show marked shifts in the frequency of the peripheral blood B cell subsets. **(a) **Dot-plots of IgD versus CD27 of peripheral blood CD19^+ ^B cells from representative healthy control and a long-standing rheumatoid arthritis (RA) patients illustrating the differences in the frequency of each B cell subset. **(b) **Box-plots representing the 10th, 25th, 50th (median), 75th and 90th percentiles of the frequencies of the total B cells (as a percentage of lymphocytes), total CD27^+ ^memory B cells, naïve IgD^+ ^CD27^- ^B cells, pre-switch IgD^+ ^CD27^+ ^memory B cells and post-switch IgD-CD27+ memory B cells (each as a percentage of B cells) in the peripheral blood of healthy donors (n = 40, white bars) and long-standing RA patients (n = 33, grey bars). *Significant (*P *< 0.01) difference from control donors.

The frequencies of B cell subsets defined by the expression of IgD and CD27 in the peripheral blood of patients with long-standing RA were compared with healthy donors (Figures 1a, b). One striking finding was that the subjects with long-standing RA had a significantly (*P *= 0.0031) lower frequency of IgD^+^CD27^+ ^pre-switch memory B cells than the healthy donors (median RA 10.4 ± 1.3% vs control 15.1 ± 1.1%). This significant difference (*P *= 0.0036) was maintained when analysing the absolute number of pre-switch memory B cells (median RA: 13.8 ± 4.7 cells/μl vs control: 21.3 ± 3.9 cells/μl). On the other hand, the frequency – but not the absolute numbers – of the IgD^-^CD27^+ ^post-switch memory population was significantly (*P *= 0.0101) increased in subjects with long-standing RA when compared with the control individuals (median RA 19.6 ± 2.9% vs control 13.2 ± 1.0%). Interestingly, no significant difference could be seen between RA patients and controls in the frequency or absolute number of the total CD27^+ ^memory B cell pool (median RA 31.3 ± 3.8% vs control 30.3 ± 1.6%, *P *= 0.6258; median RA 41.0 ± 11.3 cells/μl vs control: 44.6 ± 5.0 cells/μl, *P *= 0.7022). Finally, the frequency of IgD^+^CD27^- ^naïve B cell population in the peripheral blood of subjects with long-standing RA was comparable with the healthy donors (median RA 57.3 ± 4.1% vs control 65.6 ± 1.7%). However, the absolute number of naïve B cells was significantly (*P *= 0.0231) lower in the long-standing RA patients when compared with the control individuals (median RA: 61.4 ± 28.6 cells/μl vs control: 100.5 ± 10.7 cells/μl). These differences could not be the result of B cell lymphopenia, because the absolute number of the total B cell pool in the long-standing RA patients was comparable to the healthy donors (median RA 151.0 ± 35.8 cells/μl vs control: 148.5 ± 20.0 cells/μl).

In order to assess whether disease duration had an influence on the B cell subset disparities between RA patients and healthy individuals, the frequencies of the different B cell subpopulations in the RA patients with long-standing disease (natural history patients, median disease duration 13 years) were compared with the baseline values of another group of patients with shorter disease duration (median disease duration 4.4 years) who had enrolled in a clinical trial examining the impact of MTX versus that of the combination of MTX and infliximab (Table 1). Both groups had comparable frequencies and absolute numbers of pre-switch memory B cells (median shorter disease: 10.2 ± 1.5% vs long-standing disease: 10.4 ± 1.3%, *P *= 0.8156; median shorter disease 14.4 ± 2.9 cells/μl vs long-standing disease: 13.8 ± 4.7 cells/μl, *P *= 0.4003), each of which was significantly (*P *< 0.03) lower than that found in the healthy controls. Notably, however, the frequency of post-switch memory B cells was significantly (*P *= 0.0025) lower in the patient group with shorter disease duration when compared with the long-standing group (median shorter disease: 9.7 ± 2.9% vs longer standing disease: 19.6 ± 2.9%). Furthermore, the frequency of the total CD27^+ ^memory B cell population was significantly (*P *= 0.0184) lower in the patient group with shorter disease duration when compared with the long-standing group (median shorter disease: 19.1 ± 3.2% vs longer standing disease: 31.3 ± 3.8%).

### Reduced peripheral blood pre-switch IgD^+^CD27^+ ^memory B cell population is not the result of CD27 shedding

Shedding of surface CD27 from peripheral blood pre-switch memory B cells could account for the reduced frequency of IgD^+^CD27^+ ^B cells in RA patients. Moreover, CD27^- ^memory B cells have been recently reported in healthy individuals [14,16] and in SLE patients [15]. Therefore, to verify whether the IgD^+^CD27^- ^B cells in RA patients were actually naïve, single-cell PCR analysis of immunoglobulin heavy chain variable region (IgV_H_) genes from sorted peripheral blood, IgD^+^CD27^+ ^and IgD^+^CD27^- ^RA B cells, was carried out to determine the frequency of somatic mutations in those subsets. As expected, most of the IgD^+^CD27^- ^B cells expressed unmutated IgV_H _genes (76%) and those that were mutated contained few mutations. Both the frequency of mutated Ig sequences and the mutational frequency in the IgD^+^CD27^- ^subset was significantly (*P *< 0.05) lower when compared with the IgD^+^CD27^+ ^subset. Moreover, the mean number of somatic mutations per IgV_H _gene was significantly (*P *< 0.05) lower in the IgD^+^CD27^- ^subset (Table 2). Thus, there was no evidence that the IgD^+^CD27^- ^population of RA patients contained a subgroup of pre-switch memory B cells that failed to express CD27.

**Table 2:** Mutational frequencies of B cell subsets in peripheral blood from patients with rheumatoid arthritis

Population	Number of Sequences	Frequency (number) of mutated sequences	Mean (range) number of mutated nucleotides per mutated sequence	Overall mutational frequency	Mutational frequency for mutated sequences
CD19^+ ^IgD^+ ^CD27^-^	92	24%^a ^(n = 22)	1.8 bp^a ^(1 to 5 bp)	1.7 × 10^-3a^	7.3 × 10^-3a^
CD19^+ ^IgD^+ ^CD27^+^	28	96% (n = 27)	12.3 bp (2 to 29 bp)	4.9 × 10^-2^	5.1 × 10^-2^

^a ^Significant difference (*P *< 0.05) between the CD19^+ ^IgD^+ ^CD27 and the CD19^+ ^IgD^+ ^CD27^+ ^subsets.

To confirm that CD27 shedding from the surface of memory B cells was unlikely to be responsible for the reduction of the pre-switch memory B cell population in the peripheral blood of RA patients, the levels of soluble CD27 in the serum of RA patients and control individuals were determined by ELISA. As other studies have previously reported [31], no differences could be detected between healthy donors and RA patients (data not shown).

### CD27^+ ^memory B cells accumulate in the synovial membrane of RA patients

**Figure 2:**
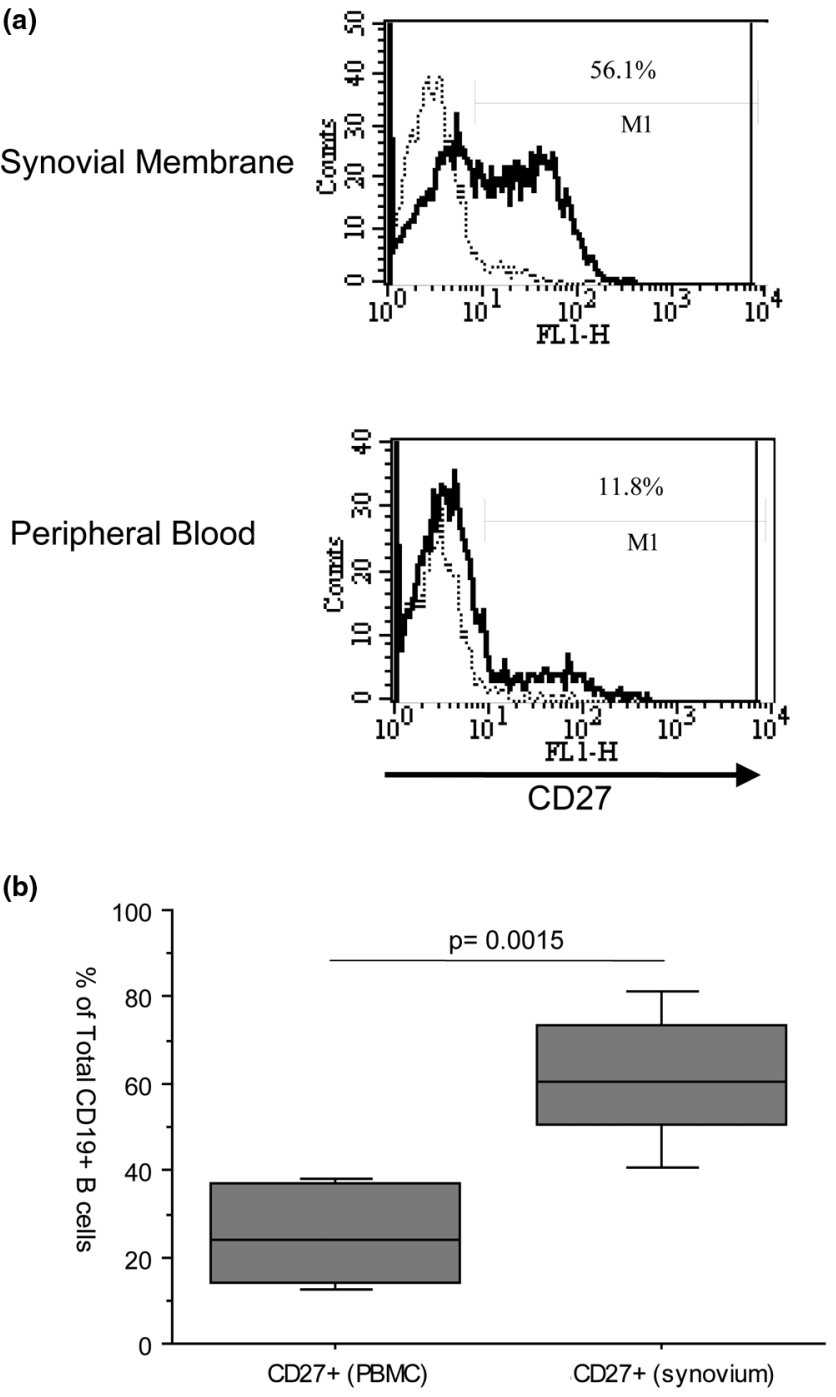
CD27^+ ^memory B cells tend to accumulate in the synovial membrane of RA patients. **(a) **Histogram from a representative rheumatoid arthritis (RA) patient showing the difference in CD27 expression in peripheral blood and synovial CD19^+ ^B cells. Dashed line shows staining with the isotype control and the solid line for CD27. **(b) **Box-plots representing the 10th, 25th, 50th (median), 75th and 90th percentiles of the frequency of CD27 expression by peripheral blood and synovial CD19^+ ^B cells in long-standing RA (n = 10).

It is known that the rheumatoid synovial membrane is infiltrated by B and plasma cells [3,6]. In order to determine the nature of the B cell subsets that comprise the synovial B cell population in long-standing RA, lymphocytes isolated from the synovial membrane and from peripheral blood of RA patients with long-standing disease were phenotyped by flow cytometry. As depicted in Figure 2a for one representative patient, the majority of the synovial B cells express CD27. In this patient cohort the rheumatoid synovial membrane had significantly more CD27-expressing B cells than the peripheral blood (61.4 ± 10.4% vs 25.1 ± 15.6% respectively; *P *< 0.0001; Figure 2b). Both pre-switch IgD^+^IgM^+ ^memory cells and post-switch IgD^-^IgM^- ^memory cells were found in the synovial tissue (data not shown).

### RA peripheral blood and synovial memory B cells express abnormal chemokine receptor patterns

Chemokine receptor expression by RA B cells can be indicative of their preferential capability for homing into the inflamed tissues. To assess whether the expression of chemokine receptors by RA synovial CD27^+ ^memory B cells could contribute to the skewed distribution of the different B cell subsets in the RA synovial membrane, the frequencies of CD27^+ ^memory B cells expressing specific chemokine receptors was also determined. Notably, expression of CXCR1, CXCR2, CXCR4 and CCR2 was significantly elevated in synovial compared with blood memory B cells (*P *< 0.00001) of either controls or RA patients (Table 3). Moreover, RA blood B cells manifested significantly enhanced expression of CXCR1, CXCR2 and CCR2 and decreased expression of CXCR4 compared with control blood (*P *< 0.02).

**Table 3:** Chemokine receptor expression by CD19^+ ^CD27^+ ^memory B cells from peripheral blood of healthy donors and RA patients and in RA synovium

	**Peripheral blood**		**Synovium**
		
**Chemokine receptor**	Control (n = 13)	RA (n = 20)	(n = 10)
**CXCR1**	5.0 ± 0.6%	16.9 ± 1.2%^a^	39.0 ± 2.2%^b^
**CXCR2**	3.4 ± 0.4%	7.7 ± 0.6%^a^	31.2 ± 1.6%^b^
**CXCR4**	60.0 ± 0.9%	47.5 ± 2.3%^a^	92.1 ± 0.7%^b^
**CCR2**	2.8 ± 0.4%	7.6 ± 0.6%^a^	28.7 ± 1.8%^b^

^a ^indicates significant difference (*P *< 0.02) between controls and patients with rheumatoid arthritis (RA);

^b ^indicates significant difference (*P *< 0.00001) between RA blood and synovium.

Data are means ± standard error of the mean.

### Anti-TNF therapy increases the pre-switch IgD^+^CD27^+ ^memory B cell population

As shown in Table 4, the group of patients receiving infliximab plus MTX therapy exhibited significant (*P *< 0.05) improvement of several laboratory and clinical parameters, including disease activity score, rheumatoid factor titres, ESR and the number of swollen and tender joints. By contrast, after the sixth treatment, the group receiving MTX monotherapy manifested significant (*P *< 0.05) improvement only in disease activity score and in the number of tender joints.

**Table 4:** Laboratory and clinical parameters of the patients undergoing MTX or MTX plus infliximab therapy at baseline (first visit) and after six treatments (seventh visit)

	MTX (n = 8)		MTX + infliximab (n = 15)	
		
	first visit	seventh visit	first visit	seventh visit
DAS 44	5.2 ± 0.4	4.4 ± 0.4*	5.8 ± 0.5	3.9 ± 0.5*
RF (IU)	210.1 ± 117.9	146.4 ± 80.4	396.2 ± 178.4	257.3 ± 142.8*
CRP (mg/dl)	0.8 ± 0.3	0.6 ± 0.2	1.8 ± 0.5	1.1 ± 0.3
ESR (mm/hour)	36.9 ± 5.4	31.9 ± 5.0	60.2 ± 8.1	44.2 ± 5.5*
SJC	19.6 ± 4.6	14.0 ± 3.0	21.9 ± 3.1	11.6 ± 2.4*
TJC	25.8 ± 4.9	16.0 ± 4.3*	27.0 ± 4.5	12.1 ± 3.4*

Data are means ± standard error of the mean.

* *P *< 0.05 between first and seventh visit.

CRP = C-reactive protein; DAS = disease activity score; ESR = erythrocyte sedimentation rate; MTX = methotrexate; RF = rheumatoid factor; SJC = swollen joints count; TJC = tender joints count.

**Figure 3:**
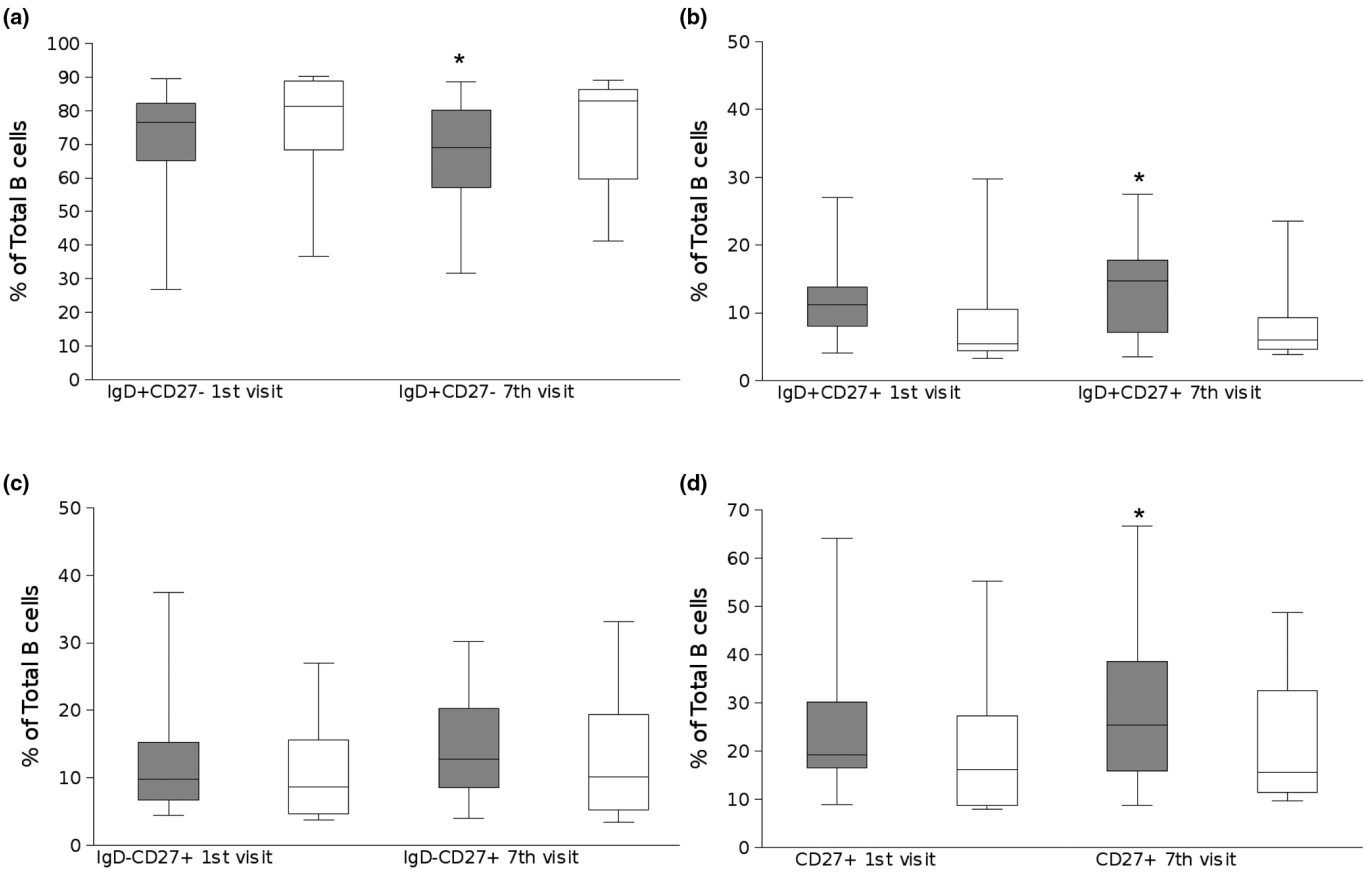
TNF blockade induces an increase in the frequency of peripheral blood total memory and pre-switch memory B cells, while reducing the circulating naïve B cells. **(a) **Box plots representing the 10th, 25th, 50th (median), 75th and 90th percentiles of the frequency of IgD^+ ^CD27^- ^naïve B cells at the time of the 1st and 7th visits for the patients in the infliximab plus methotrexate (MTX) group (n = 15, grey bars) and MTX monotherapy (n = 8, white bars). **(b) **Box plots representing the 10th, 25th, 50th (median), 75th and 90th percentiles of the frequency of IgD^+ ^CD27^+ ^pre-switch memory B cells at the time of the 1st and 7th visits for the patients in the infliximab plus MTX group (n = 15, grey bars) and MTX monotherapy (n = 8, white bars). **(c) **Box plots representing the 10th, 25th, 50th (median), 75th and 90th percentiles of the frequency of IgD^- ^CD27^+ ^post-switch memory B cells at the time of the 1st and 7th visits for the patients in the infliximab plus MTX group (n = 15, grey bars) and MTX monotherapy (n = 8, white bars). **(d) **Box plots representing the 10th, 25th, 50th (median), 75th and 90th percentiles of the frequency of total CD27^+ ^memory B cells at the time of the 1st and 7th visits for the patients in the infliximab plus MTX group (n = 15, grey bars) and MTX monotherapy (n = 8, white bars). * Significant (*P *< 0.01) difference from 1st visit.

In order to determine whether anti-TNF therapy had an effect on the distribution of the different peripheral blood B cell subsets in RA, the frequencies of those subsets were calculated after six administrations of study drug (Figure 3). At the time of the first visit all the RA patients in both treatment groups had comparable frequencies of IgD^+^CD27^- ^naïve B cells, IgD^+^CD27^+ ^pre-switch memory B cells and IgD^-^CD27^+ ^post-switch B cells. Before any of the therapies had been initiated all RA patients and healthy controls had a comparable frequency of naïve B cells (median controls: 65.6 ± 1.7% vs infliximab + MTX: 76.5 ± 4.4%; MTX: 81.4 ± 6.7%). Similarly, the frequency of the post-switch memory B cell population in all RA patients before treatment was comparable to healthy donors (median control: 13.6 ± 1.0% vs infliximab + MTX: 9.7 ± 2.6%; MTX: 8.7 ± 3.4%), whereas the pre-switch memory B cell subset was significantly (*P *< 0.03) lower in RA patients than in the control individuals (median control: 15.1 ± 1.1% vs infliximab + MTX: 11.2 ± 1.5%; MTX: 5.4 ± 3.1%).

When compared with the baseline values from the first visit, the RA patients receiving infliximab plus MTX significantly (*P *< 0.01) increased the frequency of total and pre-switch peripheral memory B cells after the sixth round of treatment (median baseline 11.2 ± 1.5% vs after treatment 14.7 ± 1.8%) However, no changes could be observed in the patients receiving MTX alone (median baseline 5.4 ± 3.1% vs after treatment 5.9 ± 2.4%).

In order to determine whether the changes observed after TNF-blockade exclusively involved B cells, the frequencies of CD4 and CD8 T cells were analysed in both patient groups. However, no changes were observed between visits in either treatment group (data not shown).

## Discussion

Abnormal distributions of peripheral B cell subsets, particularly of CD27^+ ^memory B cells, have been reported in several autoimmune diseases including RA [15-18,20,21]. However, in RA there is no consensus about the nature of the abnormalities, because some reports note an increase and others no change in peripheral memory B cells [19-21]. In the present study, we report that RA patients have a lower frequency of circulating pre-switch IgD^+^CD27^+ ^memory B cells when compared with healthy individuals. Importantly, memory B cells accumulate in the synovial membrane of subjects with RA, suggesting that accumulation of pre-switch memory B cells within inflamed tissue may contribute to a decrease in this B cell subset in the blood. It should be pointed out that post-switch IgD^-^CD27^+ ^memory B cells are also enriched in the rheumatoid synovium, although these cells are not decreased in the blood in early RA. This discrepancy may be explained by the more complex homeostasis of post-switch memory B cells. Although these cells accumulate in the synovium, they are also generated in increased numbers in patients with RA [19]. As a result, post-switch memory B cells accumulate not only in the synovium but also in the blood of patients with long-standing RA.

The human memory B cell population is heterogeneous, comprising mutated pre-switched IgD^+^CD27^+ ^and post-switch IgD^-^CD27^+ ^B cells [13,32], that develop with age [33]. It is widely accepted that post-switch IgD^-^CD27^+ ^memory B cells are post-germinal centre highly mutated memory B cells [11,13,32,34]. However, the function and the origin of the pre-switch IgD^+^CD27^+ ^subpopulation is still a matter of controversy. Despite some characterisation [35], it has not been clearly established whether the IgD^+^CD27^+ ^memory population only participates in T cell-independent immune responses, because this population expresses heavily mutated Ig genes [32]. The role of this population in autoimmune diseases has been stressed by the finding that in the peripheral blood of SLE and SS patients the pre-switch memory B cell subset is markedly reduced [18,36]. However, in RA patients with long-standing disease, previously published data suggested that there might be an accumulation of CD27^+ ^memory cells in the peripheral blood [20,21], and especially of the post-switch IgD^-^CD27^+ ^memory subset, whereas the IgD^+^CD27^+ ^subset was reported to be comparable to healthy donors [20]. We were unable to confirm these findings. Instead, we observed that patients with RA manifested a marked reduction in the peripheral blood pre-switch IgD^+^CD27^+ ^subset. At baseline the group of patients engaged in the clinical trial and those with shorter disease duration had similarly lower frequencies and absolute numbers of peripheral blood pre-switch IgD^+^CD27^+ ^memory B cells compared with the group of patients with long-standing disease, so this abnormality would seem to be an integral feature of RA, independent of disease duration. Preliminary assessment of a group of patients with very early RA, who had disease duration of less than six weeks and had received no DMARD therapy, also indicated a decrease in IgD^+^CD27^+ ^pre-switch memory B cells and is consistent with the conclusion that this abnormality in memory B cell homeostasis is characteristic of RA independent of disease duration and DMARD therapy (R Moura and JE Fonseca, unpublished data). Notably, in none of the analysed RA patient groups did we observe an increase in the total CD27^+ ^memory B cells when compared with control subjects. Nevertheless, with long-standing disease in both the National Institutes of Health and Japanese cohorts, the post-switch IgD^-^CD27^+ ^population was increased. This is likely to be related to the increased production of post-switch memory B cells owing to persistent immunological stimulation that is sufficient to overcompensate for the enhanced sequestration of these cells in the synovium.

The disparities between our data and the results previously reported [19-21] may be explained by a number of factors, including disease duration, cohort size and therapy. Importantly, most studies did not analyse patients in terms of disease duration, which as we report here can clearly affect memory B cell subset distribution. It is notable that when total CD27^+ ^memory B cells were analysed in patients receiving only MTX therapy, a remarkably broad range of distributions was noted, with some patients with very high and others with low frequencies [19]. Finally, some studies did not separately analyse the patients on TNF-blockers, which can alter peripheral blood memory subset distribution as found here and reported previously [19].

Elevated concentrations of matrix metalloproteinases, with the capacity to cleave molecules of the TNF-family from the cell surface [37], have been reported in RA synovial fluid [38], and soluble CD27 is increased in the synovial fluid but not in the serum of RA patients [31]. Therefore, it was possible that the reduction of IgD^+^CD27^+ ^B cells in RA patients could be the result of proteolytic cleavage of CD27 (a TNF-receptor family member [39]) from the cell surface of pre-switch memory B cells. However, the negligible number of somatic mutations in the V_H _genes of the IgD^+^CD27^- ^subset and the comparable serological levels of soluble CD27 in RA patients and healthy individuals discounted the possibility that CD27 had been cleaved from the cell surface of pre-switch IgD^+^CD27^+ ^memory B cells, therefore giving them a false-naïve phenotype.

Chemokine receptor imbalances have been reported in several autoimmune diseases [21,40-43]. Additionally, several studies have provided strong evidence that in RA synovium and synovial fluid, monocytes/macrophages, synovial fibroblasts, FDC and mast cells have an increased expression of either chemokines or their receptors responsible for B and T cell recruitment [4,44-48]. Together with an accumulation of both subsets of memory B cells in the synovial membrane of RA patients with reduced peripheral blood IgD^+^CD27^+ ^B cells, we have also observed significant shifts in the expression of several chemokine receptors in the RA peripheral blood B cell subsets and in the synovial memory B cells: the frequency of CD27^+ ^memory B cells expressing the pro-inflammatory CXCR1, CXCR2 and CCR2 chemokine receptors [49,50] was elevated in both peripheral blood and synovium; and contrary to RA peripheral blood, the large majority of synovial membrane memory B cells expressed CXCR4. The high frequency of RA peripheral blood and synovial membrane memory B cells expressing pro-inflammatory chemokine receptors, such as the IL8-receptors CXCR1 and CXCR2, or CCR2 (a negative modulator of cytoskeleton rearrangement and immature B cell migration [51]), stresses the potential role of interactions of memory B cells with other effector cells of the immune system that could contribute to the perpetuation of chronic synovitis. An important, and novel, finding was the abnormally increased frequency of CXCR4^+ ^memory B cells in the RA synovium. CXCR4 is the receptor for the homeostatic and pro-inflammatory chemokine CXCL12 and is expressed by mature naïve B cells when they recirculate through germinal centres of secondary lymphoid organs [52]. CXCR4 expression is essential for correct formation of the dark and light zones of the germinal centre [53]. Moreover, CXCR4 is involved in plasma cell function, because mice lacking CXCR4 expression present major abnormalities in plasma cell homeostasis [54], whereas human peripheral blood CD27^+ ^memory B cells increase CXCR4 expression upon differentiation into plasma cells [55]. The importance of CXCR4 expression in synovial membrane inflammation has been emphasised by the inhibition of collagen-induced arthritis by the CXCR4 antagonist T140, and by the finding of elevated CXCR4 gene expression in RA synovial biopsies with follicular-like lymphoid structures [4]. Therefore, our data are consistent with the possibility that in RA elevated numbers of CXCR4^+ ^memory B cells may be recruited into the synovial membrane where they accumulate, and might be involved in seeding follicular-like structures and/or differentiating into autoantibody-secreting plasma cells, thus perpetuating the chronic synovitis.

Anti-TNF therapy in RA has been linked to a reduction of B cells expressing the early activation marker CD23 in the peripheral blood [56]. Neutralising TNF in RA diminishes the production of pro-inflammatory cytokines in the joints, lowers the levels of circulating IL1, IL6 and acute-phase proteins [22,25], and decreases the serological levels of ICAM-1, ICAM-3, VCAM-1, VEGF and E-selectin [57,58]. In the synovium, infliximab induces a major reduction of sublining T cells, B cells and macrophages [59], decreases ectopic lymphoid neogenesis [5], and lowers the expression of IL8 and MCP-1/CCL2 [60]. A major finding in the present study was the normalisation of the peripheral blood pre-switch memory B cell population in RA patients who received anti-TNF therapy, which was accompanied by a significant amelioration of several clinical parameters. This recovery of circulating pre-switch memory B cells after infliximab treatment contrasts with the findings in a recent report using etanercept [19]. By blocking TNF and LTα simultaneously with etanercept, the possible effects of LTα on B cell homeostasis and GC formation make it difficult to identify the individual contribution of TNF-blockade on the B cell compartment in RA. As a result of treating a group of RA patients with infliximab, (that specifically blocks TNF) we have found an increase in circulating pre-switch memory B cells. It is difficult to be certain whether the increase in pre-switch memory B cells specifically related to an improvement in disease activity or specifically the blockade of TNF. In this regard, the patients who received only MTX therapy exhibited some improvement in clinical disease activity, but it was not as profound as that noted in patients treated with MTX and infliximab. Therefore, it is uncertain whether the lack of significant change in circulating pre-switch memory B cells in the patients receiving MTX resulted from a failure to block TNF completely or from an incomplete clinical response. The effect of TNF blockade, however, did appear to be specific for pre-switch memory B cells. Although, previous studies reported a transient increase in T cell counts after four weeks [61] and after repeated doses [62] of anti-TNF therapy, we could not detect any significant changes in any of the T cell populations in the patients treated with the combination of infliximab and MTX. Therefore, these results suggest that neutralising TNF alone might block the migration of memory B cells into the synovium without a persistent effect on T cell trafficking, which support recent findings showing that in RA patients with disease remission after anti-TNF therapy (using either specific TNF blockers or simultaneous blockade of TNF and LTα) the number and the density of B cells in the synovial membrane and the frequency of ectopic germinal centres significantly decreased [5].

## Conclusions

In summary, the present findings indicate that the reduction of circulating memory B cells in RA patients, particularly the pre-switch memory subset, might be linked to their accumulation in the inflamed rheumatoid synovium under the influence of TNF.

## Abbreviations

APC: allophycocyanin; BSA: bovine serum albumin; CRP: C-reactive protein; DMARD: disease-modifying anti-rheumatic drugs; DMEM: Dulbecco's Modified Eagle's Medium; ELISA: enzyme-linked immunosorbent assay; ESR: erythrocyte sedimentation rate; FCS: fetal calf serum; FDC: follicular dendritic cell; FITC: fluorescein isothiocyanate; ICAM: intercellular adhesion molecule; IgV_H_: immunoglobulin heavy chain variable region; IL: interleukin; LT: lymphotoxin; MAb: monoclonal antibodies; MTX: methotrexate; PBMC: peripheral blood mononuclear cells; PBS: phosphate-buffered saline; PCR: polymerase chain reaction; PE: phycoerythrin; PerCpCy5.5: peridinin-chlorophyll-protein Cy5.5; RA: rheumatoid arthritis; SLE: systemic lupus erythematosus; SS: Sjögren's syndrome; TNF: tumour necrosis factor; VCAM: vascular cell adhesion molecule; VEGF: vascular endothelial growth factor.

## Competing interests

The authors declare that they have no competing interests.

## Authors' contributions

MMS-C collected data, designed experiments, carried out statistical analysis and wrote the manuscript. VM carried out the clinical study and collected data. RG-M designed and carried out the clinical study. RF, KT, TN, MW, MB and TAF collected patient data. PEL designed and coordinated the study and wrote the manuscript.
